# Iron management and exercise training in individuals with chronic kidney disease: lived experiences

**DOI:** 10.1093/ckj/sfae433

**Published:** 2025-01-03

**Authors:** Courtney J Lightfoot, Sharlene A Greenwood, Elham Asgari, Debasish Banerjee, Sunil Bhandari, James O Burton, Philip A Kalra, Kieran McCafferty, Benjamin A Oliveira, Chante Reid, Pauline A Swift, David C Wheeler, Thomas J Wilkinson, Kate Bramham, Alice C Smith

**Affiliations:** Department of Population Health Sciences, University of Leicester, Leicester, UK; NIHR Leicester Biomedical Research Centre, Leicester, UK; King's College Hospital NHS Foundation Trust, London UK; King's College London, London, UK; Guy's and St Thomas’ NHS Trust, London, UK; St George's University Hospitals NHS Foundation Trust, London, UK; Hull University Teaching Hospitals NHS Trust, Hull, UK; Department of Cardiovascular Sciences, University of Leicester, Leicester, UK; John Wall's Renal Unit, University Hospitals of Leicester NHS Trust, Leicester, UK; Salford Royal Hospital, Northern Care Alliance NHS Foundation Trust, Salford, UK; Barts Hospitals NHS Trust, London, UK; King's College London, London, UK; King's College Hospital NHS Foundation Trust, London UK; Epsom and St Helier University Hospitals NHS Trust, London, UK; University College London, London, UK; Department of Population Health Sciences, University of Leicester, Leicester, UK; NIHR Leicester Biomedical Research Centre, Leicester, UK; Leicester Diabetes Centre, Leicester, UK; King's College Hospital NHS Foundation Trust, London UK; King's College London, London, UK; Department of Population Health Sciences, University of Leicester, Leicester, UK; NIHR Leicester Biomedical Research Centre, Leicester, UK

**Keywords:** chronic kidney disease, exercise, fatigue, iron, quality of life

## Abstract

**Background:**

Non-anaemic iron deficiency is highly prevalent in people living with chronic kidney disease (CKD) but is underdiagnosed and undertreated, especially in earlier stages of CKD. A multicentre trial assessing the effect of intravenous iron supplementation in iron-deficiency but not anaemic people with CKD included a qualitative sub-study that aimed to explore the patient experience and psychosocial impact of living with CKD and iron deficiency, and the experience of the therapeutic intervention (intravenous iron and exercise).

**Methods:**

Semi-structured interviews were conducted with 23 trial participants blinded to treatment. Topics explored included experiences of living with CKD and iron deficiency, symptoms, social and leisure activities, quality of life, and participants’ views and experiences of receiving the therapeutic intervention. Thematic analysis was used to identify and report themes.

**Results:**

Six overarching themes were identified: lack of awareness of iron deficiency; overwhelming feelings of tiredness; feeling limited; balancing emotions; perceptions and experiences of therapeutic treatment received; and impact of trial participation on life participation. Trial participation, specifically the exercise training, was perceived to be beneficial, with improvements in life participation and psychological wellbeing experienced. However, there were no clear differences between treatment groups, with mixed perceptions about which therapeutic treatment was received.

**Conclusions:**

The impact of tiredness on individuals with CKD is profound and can result in reduced vitality, impaired ability to engage in life activities and emotional conflict. Improved communication and support about psychosocial impact and management of symptoms, particularly fatigue, for people with CKD may be required, alongside effective therapeutic interventions, to improve symptom management and quality of life.

KEY KEY LEARNING POINTS **What was known:**

- Iron-deficient anaemia is a well-recognized association and complication of chronic kidney disease (CKD); non-anaemic iron deficiency is more prevalent but is underdiagnosed and treated, especially in earlier CKD stages.
- Intravenous iron in non-dialysis CKD is not routinely offered, and selection for treatment is based on the patient's needs, but there is increased support for its use in CKD stages 3–5.
- Understanding the patient's perspective and experience is an essential part of the evaluation of therapeutic strategies.

**This study adds:**

- Overwhelming feelings of tiredness and substantial psychosocial and physical burden associated with living with CKD were frequently experienced by participants.
- There were no clear differences reported between treatment groups, with mixed perceptions about which therapeutic treatment was received.
- Study participation was perceived to be beneficial with both psychological and physical improvements reported; however, it was not possible to differentiate or attribute improvements to specific components of the trial (e.g. iron therapy, placebo effect, exercise training).

**Potential impact:**

- The findings suggest that improved communication and support about the psychosocial impact and management of symptoms, particularly fatigue, for people with CKD may be required, alongside effective therapeutic interventions, to improve symptom management and quality of life.

## INTRODUCTION

Iron, an essential mineral, is critical for numerous metabolic processes within the body [1, 2]. Iron deficiency can occur due to low availability as a result of reduced intake/absorption, increased usage/loss, or inability to mobilize and utilize iron stores within the reticuloendothelial system [3, 4]. Iron deficiency classically presents and is diagnosed as iron-deficiency anaemia [5]; however, an iron-deficient state precedes overt anaemia [6], and is associated with wide-ranging signs and symptoms [7], including weakness and fatigue, poor concentration and low exercise tolerance [8].

Most people with chronic kidney disease (CKD) live with stable or slowly declining kidney function associated with high comorbidity and symptom burden, which often includes dysregulated metabolic and immune function [9, 10]. Although iron-deficiency anaemia is a well-recognized complication of CKD [11], non-anaemic iron deficiency is much more prevalent but is underdiagnosed and treated, especially in earlier CKD stages [12]. Consequences include overwhelming fatigue, reduced exercise capacity and poor physical function, as well as increased risk of morbidity and mortality [13, 14], and decreased quality of life (QoL) [15]. Impaired QoL is common in people living with CKD, with almost three-quarters experiencing problems in at least one dimension of the EQ-5D [16].

Treatment for iron deficiency involves iron supplementation which can be administered orally or intravenously [17]. Iron administration can increase haemoglobin levels in people with CKD and anaemia [18]. Whilst the use of iron replacement therapy to relieve anaemia is well-established, the potential effects of correcting iron deficiency in the absence of anaemia have only recently received attention [19]. Intravenous iron supplementation is considered the gold standard for people on long-term haemodialysis, however its use in non-dialysis CKD is less definite [20]. Clinical guidelines are generally steered by measurable clinical outcomes rather than the needs or QoL of individuals [21]. Intravenous iron in non-dialysis CKD is not routinely offered, and selection is based on the patient's clinical needs [22], but there is increased support for its use in CKD stages 3–5 [20]. However, despite the evidence of iron to assist with the correction of anaemia in CKD, further evaluation of its impact in this population is required [23].

To assess the effect of intravenous iron supplementation in iron-deficient but not anaemic people with CKD, a multicentre prospective double-blind placebo-controlled randomized controlled trial was conducted [Iron and Muscle (I&M) trial] [24]. Understanding the patient's perspective and experience is an essential part of the evaluation of therapeutic strategies; thus, a qualitative sub-study was included in the I&M trial, which aimed to understand the patient experience of living with CKD and iron deficiency, and the therapeutic intervention (intravenous iron and exercise).

## MATERIALS AND METHODS

### Study design

**Figure 1:**
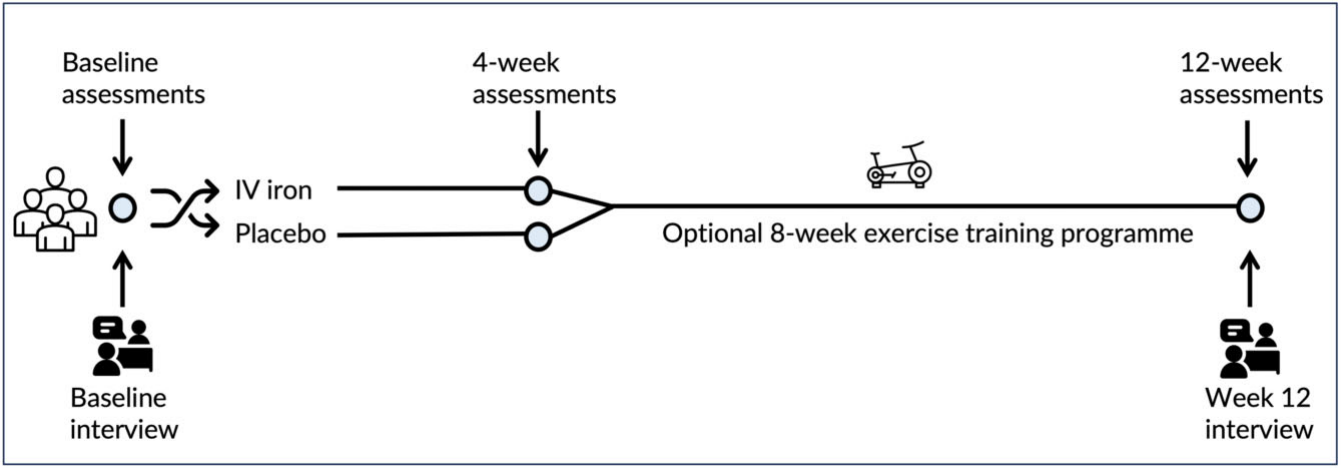
Schematic showing flow of participants through I&M trial and location of interviews during trial.

A qualitative interview sub-study was conducted within the I&M trial, a prospective, double-blind multicentre randomized controlled trial (EudraCT: 2018–000 144–25). The I&M trial aimed to ascertain whether intravenous iron therapy is beneficial to exercise capacity, muscle metabolism, physical function, fatigue and QoL in CKD, and whether iron repletion enhances the effect of an exercise intervention. In summary, over 12 weeks, in people with non-dialysis CKD who had iron deficiency without anaemia, the I&M trial examined (i) the efficacy of intravenous iron supplementation at 4 weeks; and (ii) the added efficacy of an additional optional 8-week exercise training programme (Fig. 1) [24]. Details regarding the trial processes and procedures, including specifics of exercise regimen, are published elsewhere [24, 25]. The I&M trial primary findings, describing the effects of intravenous iron and the additional exercise training programme on patient outcomes, have been published [25].

### Participants

Participants were recruited from the main I&M trial, and thus met the eligibility criteria for participation: (i) aged ≥18 years; (ii) established CKD (stages 3–4); and (iii) iron-deficient but not anaemic. Participants were invited to participate in the optional qualitative sub-study by research staff who obtained consent and recruited them to the main trial. Informed consent for the sub-study was included in the main trial consent form.

### Ethics approval and consent to participate

The protocol and related documents were approved by Brent Research Ethics Committee, UK (REC 19/LO/0128), the Health Research Authority and the UK Medicines and Healthcare products Regulatory Agency (MHRA). The study was prospectively registered on 28/01/2019 (EudraCT 2018-000144-25). All methods were carried out in accordance with relevant guidelines and regulations. Informed consent was obtained from all subjects and/or their legal guardian(s).

### Sampling

The qualitative sub-study target recruitment was 20–30 participants. This sample size was pragmatically chosen based on available resources and information power (an established concept to guide adequate sample size for qualitative studies) [26]. Participants were convenience sampled from those recruited to the main trial.

### Setting

Semi-structured interviews were conducted either in person, in a private quiet room at the participant's local hospital, or via telephone, depending on participant preference. Interviews were conducted by an experienced qualitative researcher (C.J.L.) who had no prior relationship with participants, nor involvement in any other part of the I&M trial to encourage freedom of expression. Both the qualitative researcher and trial participants were blinded to the therapeutic treatment received (intravenous iron or placebo).

### Interview procedure

Data were collected through semi-structured interviews, either face-to-face or via telephone. Participants were interviewed at baseline (i.e. prior to Ferric carboxymaltose (Ferinject^®^) or placebo administration) and at the end of the trial. Interviews were conducted on the same day as a study visit at baseline and/or 12-week follow-up, or a separate visit around these time points (<7 days), depending upon participant preference. Before the interview, the researcher explained the background and interview process. Participants were reassured about the preservation of their anonymity and confidentiality and were given the opportunity to ask questions before audio-recording commenced. Interviews were expected to last approximately 60 minutes.

Topic guides were developed in consultation with patient and public involvement partners and sought to explore the lived experience of CKD, iron deficiency, physical function and fatigue, and the impact of trial participation. The following topics were covered in the interview schedule: experiences of living with CKD and iron deficiency, symptoms, social and leisure activities, QoL, and participants’ views and experiences of receiving the therapeutic intervention. Interviews were audio-recorded and transcribed verbatim. The researcher kept a reflexivity diary, documenting thoughts and comments during and following participant interviews, and during data analysis.

### Data analysis

The recorded interviews were transcribed using professional transcription services. All identifiable information, such as individual's names and personal details, were removed from completed transcripts. QSR International's NVivo12 software was used to manage and store the data, which were analysed according to the principles of interpretive thematic analysis using the approach described by Braun and Clarke [27] to identify and report themes. One researcher (C.J.L.) read the complete data set to familiarize themself with the content and identified initial codes, followed by review, revision and determination of final codes. Each script was coded, and potential themes were identified. These were reviewed and refined, and definitions of themes were determined.

### Findings

In total, 23 participants [15 female, average age 57.6 years (range 38–72), mean estimated glomerular filtration rate 30.6 (±12.4) mL/min/1.73 m^2^, haemoglobin 126.4 (±12.3) g/L, ferritin 57.1 (±36.3) µg/L, transferrin saturation (TSAT) 21.6% (±10.5)] were interviewed: 21 on entry (pre-intervention) and 17 on exit (post-intervention) of the trial; 16 participants were interviewed at both time points. Five participants interviewed at baseline were withdrawn from the trial. Of the 17 interviewed post-intervention, 5 participants received intravenous iron supplementation. All participants received the exercise training. Participant characteristics are detailed in Table 1. Interviews lasted an average of 58 (range 28–98) minutes.

**Table 1:**
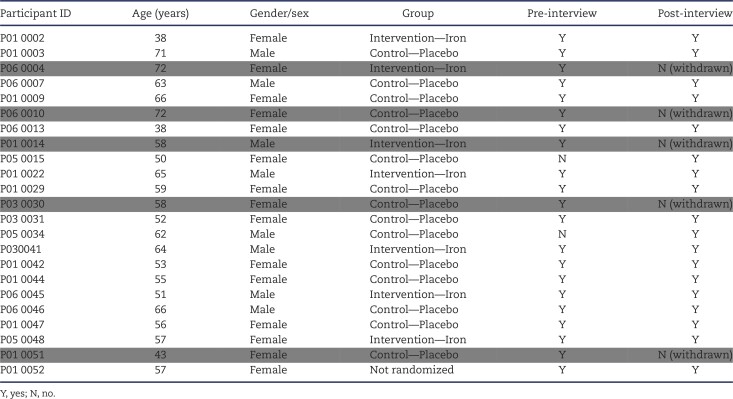
Participant characteristics.

Participants described their experiences of living with CKD and iron deficiency and the psychological and physical impact this had on them, their experiences of receiving the therapeutic interventions, and perceived effects of trial participation, specifically intervention effects (iron therapy or placebo and exercise training). Six overarching themes were identified which are displayed in Table 2.

**Table 2:** Themes and subthemes identified.

Lack of awareness of iron deficiency prior to trial involvement	Lack of awareness and understanding	‘I didn't actually know I had low iron levels before this study … and maybe if I'd known about it earlier, I might have been able to at least know why’ (Female, 67 years)
		‘My doctor never say it to me because I think the doctor probably would see it's low but it's not that low to be anaemic, so they would just dismiss it … Iron deficiency, I don't understand it, why does it do it really when your body needs’ (Male, 71 years)
		‘The iron bit is a big concern of mine because I don't really know much about it. There's no leaflets and there's no way I can communicate with people like my GP because they don't understand what's happening’ (Male, 58 years)
	Perceived consequences	‘First of all you're tired, you feel low, it can trigger other things because it depends on our mood, because if you have bad news you don't have energy to do something and if you're physically low and you can't do too much you will feel low because you can't do…!’ (Female, 56 years)
		‘You know when you're not getting enough sleep, it going to make you very tired, you get fatigue, isn't it, so I find I am very tired, right. Now if your blood count is low, according to what I hear, it gives you those symptoms too. You'll be very fatigued, you see, and tired. So what I'm trying to say again I don't know which one it would be—is it from lack of sleep or is it from low iron?’ (Male, 71 years)
	Associated symptoms experienced	‘I get quite bad cramps which is excruciatingly painful and very inconvenient in the middle of the night especially when you get cramp in both legs. And in my ankle in particular … I have I do seem to suffer increasingly from the cold and getting cold. Sometimes I get cold feet in bed and wake up with cold feet, that might have that's obviously a circulation problem’ (Male, 65 years)
		‘Sometimes I get restless legs syndrome if I get over tired, which can be a bit irritating. And I generally need to get up to go to the loo once or twice, so that's a bit annoying really. I try to take a couple of paracetamol if I think I'm a bit, a tiny bit overtired, because I know that it will only get worse’ (Female, 59 years)
		‘Just, bones hurt, body aches, but you see you don't know whether it's age now or what I've done over the years or whether it is my kidneys. You know as I say I get quite a lot of pain now just it's like that all the while’ (Female, 72 years)
Overwhelming feelings of tiredness	Perceived sensation	‘Tiredness is like—it's so hard to explain—it's like a veil, like, weighs you down, everything, your body, your mind, how you feel, how you respond, how you react to people, your general demeanour, it's just all encompassing’ (Female, 38 years)
		‘Drained, it literally feels like somebody's sort of pulled the plug out and just gone—and I feel quite achy. Physically, slightly—very, very mildy fluey. You know that feeling when the flu's just coming on and you've got that sort of heavy, very slightly achy—it's how I feel’ (Female, 66 years)
		‘When I wake up sometimes, it's like when I was younger and I'd wake up with a hangover and you just feel that kind of, urghh, some mornings it could be like that, but it passes quicker, you know, if it's a hangover it's with you for a few hours, whereas this is just like it's sort of half an hour to come round and then you accept you have to get out of bed, so you do … I'm aware I'm not feeling refreshed and I don't want to spring out of bed’ (Female, 50 years)
	Perceived causes	‘I am puzzled about why I'm so tired … I lie down, despite the fact it's not good sleep, I have to put the blame on that because I'm not sleeping well, as I say, it's just tired’ (Male, 71 years)
		‘I think I get tired because I'm just not doing more things… you know when you just sit down and do nothing? And then you get up and do something and you get tired. So I think my body's got so used to not doing anything. If I do something I get tired’ (Female, 57 years)
		‘I think a lot of the times you're just bored and you would just have a bit of a lie down, turn the radio on or reading and the next thing you fall asleep. And then you wake up in about two hours’ time and then you think because you've had that rest, you can't go to bed at the right time, so you end up staying up until about midnight/one o'clock so it's a vicious circle’ (Male, 62 years)
	Perceived consequences	‘I'm knackered all the time which is really hard and I know if it wasn't for my kidneys, I'd be, I'm only 38, I'd be sort of bouncing around but it's so debilitating because I'm so tired so much and you just want to be like fun mum that's leaping round the park and to be honest, it's took all my energies to get them ready, get them ready to go and go, and by the time you're anywhere you're like I'm knackered now which is really hard to deal with. You just want to be normal’ (Female, 38 years)
		‘Obviously you don't function well anyway …. In the morning it's about probably let's say 10. Lunchtime, probably about 6. When you have your break, you're fine because you've got things to do, so you like try to regenerate and everything like that, then obviously towards the end of the day probably about 3 or something like’ (Male, 51 years)
		‘The problem with it is that you don't know when that's going to happen. So sometimes you think oh my God, I'm not sure that I can do that because what if I wake up and feel like I'm exhausted and my bones are too heavy for my body? But I kind of try to always think that I could be like that every single day and never actually do anything ever again, so you just need to forget about it really’ (Female, 59 years)
	Exacerbated by poor sleep	‘I have no energy … I'm always tired anyway but that's basically because I don't ever get a full night's sleep … I don't know the last time I had a good night's sleep, to be quite honest with you … Whenever I feel tired, whatever time of day or night it is, as soon as I'm tired, I will go to sleep because I don't know when I'm going to get anymore sleep’ (Female, 55 years)
		‘Sometimes I can get up in the morning, have me breakfast and I can literally nod back off. If I let it …. Although if I've had a good night, I'll still get tired in the day anyway. I suppose it's better if I've had a good night's sleep’ (Female, 72 years)
	Energy management	‘If I'm just sitting, if I'm sitting at home on my own. I'm more likely to maybe drop off to sleep unless I'm like actively watching something that I'll be interested in on the tv or reading a book or something … If I've done a lot of strenuous stuff and then I sit down … If I feel tired and I've had a nap once I wake up, I'm ok. It's not like it takes me three hours to pull myself together and do whatever like. If I was to have a nap, I'll wake up and I think right ok right that's it ok. I'm ready to go now’ (Male, 63 years)
		‘It's not so much tiredness as lack of energy. I just have no energy to do anything. It's easier to do nothing than attempt to do things because I've just no energy’ (Female, 55 years)
		‘It's a bit of a drain, yeah … It's not impeding me you know. If say you had nothing the matter, you probably would fight through it … Thirty years ago, you were let's get on with it, fight through it but you can do that when you're younger. So I do try to respect, it's like an old engine, like an old car. When it was new it would do a lot more. When it gets a bit older it will still do it but you have to respect it and that's what I try to do with myself if I can’ (Male, 66 years)
Feeling limited	Impaired function activities	‘I'm probably not as strong as I used to be but that may be because I'm not necessarily I don't know picking, I find, find for instance and this may be just an age-related thing that I got to a point where I was having more difficulty in shall we say getting up out of the bath. Or getting up and down out of a chair’ (Male, 65 years)
		‘I can't do things for myself. Or anybody else … even putting stuff in the fridge, you know like they've done the shopping and my daughter tries to do things but she puts them all on the island, so I don't have to bend, just to load even the fridge, putting the food in the fridge is a chore, it's not easy’ (Female, 57 years)
		‘I can still do the same but at about 60 odd per cent slower, 70% slower … it's about pacing … I just want to be a little bit more physically capable … just want to be able to walk properly and have more strength in my legs then I think that would be half of my battle’ (Male, 64 years)
	Discordance between mind and body	‘I would like to spend some time thinking about my body and how I actually feel so that I try and differentiate actual tiredness from just feeling a bit miserable. And also how far I can push myself physically because I've given up … I just think it will give me more knowledge to understand my own body and to listen to my own body’ (Female, 53 years)
		‘I am trying not to lose energy for anything, just because if I'm physically more exhausted, that it affects me mentally more and I start crying. That's why I'm trying to do less physically but I'm not exhausted … I'm still capable. I can do more but I don't want to overload with physical stuff myself because when I'm more exhausted, and if I'm more exhausted my body probably will not manage to cope’ (Female, 56 years)
		‘You have to force yourself to more or less get up and get out, you're still tired, see, but you can't stay there all the time … I make a determination that I am not going to stay in the house or in the bed despite the fact that I'm very tired, see, so I will come out and still be moving along painfully and tired, but you won't detect it’ (Male, 71 years)
		‘Got no energy when I go the minds willing the body won't follow me. Just won't go with me. I want to do it but it just won't … I get very tired very lethargic very very lethargic all the while I want to do but can't get going. I have to push myself all the while’ (Female, 72 years)
	Constrained social life	‘I don't want people know … I didn't tell them, so they don't know … I don't go out a lot because, if I go … I don't want them to pity me’ (Male, 64 years)
		‘You don't know what to do with yourself. So I don't think you're much fun going out and invariably want to sit and chat to you when you go out and you know, you think “I just don't want to do this tonight” you know’ (Male, 66 years)
		‘I don't really have a social life anymore. I don't go out unless it's to the hospital or doctors because pain and lack of energy just stop me from doing everything basically’ (Female, 55 years)
		‘Most of them—quite a lot don't know about it … some of them are surprised. I think they think I shouldn't do as much—a lot—actually that is true—quite a few people say oh you do too much, because you've got kidneys, you know, you should do a lot less … I think they find that quite hard to understand’ (Female, 66 years)
Balancing emotions	Emotional conflict	‘It's a constant sort of balance in my head with how do I actually feel, how do I feel about how I feel and just getting through the day … [it's] very difficult to deal with’ (Female, 54 years)
		‘Let's say my wellbeing is just 20% what it was three years ago. I just feel myself really constantly unwell … It affects you mentally. If you're mentally affected, you don't have to do anything. You don't have any stimulation and it's like a vicious circle’ (Female, 56 years)
		‘I do get I get annoyed with myself not stressed or angry I just get annoyed with myself … And I get annoyed because that's stopping me from doing what I want to do. And then I get that arghhhhh. Not annoying or just ratty with myself I would say. Because …. The expectations that I put on myself’ (Male, 63 years)
		‘I get frustrated because I'm tired. Tiredness is the one thing that I can't cope with. I don't do anything … when I'm tired, you know, I'm worried and I get impatient’ (Male, 58 years)
	Low mood	‘It's a bit demoralizing, like, thinking—you sometimes feel like you know oh god what's going on, like, is it worth it, not in a bad way but, you know, I'm thinking … Makes me feel down obviously, compared to what I used to be’ (Male, 51 years)
		**‘**I wish I could have my health back, that's what I say to people, because they can do all the things that I can't and it does make me feel depressed and stuff but I don't know … I get stressed out. And I get stressed out by that but because I want do to [it]’ (Female, 52 years)
		‘First of all you're tired, you feel low, it can trigger other things because it depends on our mood, because if you have, you know, yourself, if you have bad news you don't have energy to do something and if you're physically low and you can't do too much you will feel low because you can't do! I try not to think about it. I don't know how. Not very happy but nothing they can do about me. Can't just cry about this’ (Female, 56 years)
	Coping mechanisms	‘Not wanting to do anything. My comfort zone is my bed, I close myself to everything. And being alone, it's magnified quite a bit’ (Male, 58 years)
		‘I had one of them last week ended up lying on my bed all day crying my eyes out. Because I was so bored. I just felt you know oh god if this is living. Selfish talk that … I tell myself off. Move it woman get yourself. My moto is you can only go so far down you've got to come back up’ (Female, 72 years)
Perceptions and experiences of therapeutic treatment received	Perceived therapeutic treatment received and rationale	‘I was curious as to what I got because I was hoping that with the infusion, I would have felt an improvement bodily, you understand? But the point remain is I don't know which dose I got, but I know for sure whatever it was I felt no different than before’ (Male, 71 years, Control—Placebo)
		‘I think I had the iron infusion. Because I feel different now to what I felt initially. More energetic, more like you know, with it. Not feeling down quite a lot’ (Male, 51 years, Intervention—Iron)
		‘I thought I'd worked out what it was that I had but I'm not sure I did … I suspect I had a saline drip … I don't know that I felt any different, to be truthful, which is possibly another reason why I felt as though I may have had a saline drip’ (Male, 65 years, Intervention—Iron)
		‘I am 99% convinced I got the iron—but I could also be massively influenced by placebo … I just started to feel like I had more energy in the week following that. I didn't need an afternoon sleep, whereas I always had one’ (Female, 53 years, Control—Placebo)
		‘For the first couple of weeks I was convinced that I'd been given the placebo … Because I was still had a lot of muscle pain, that I didn't feel any different. And then it wasn't until about three weeks ago when I started getting a lot of muscle pain again that I suddenly realised that I'd spent about three or four weeks with no muscle pain. I didn't even notice, but I'm now guessing that I actually got the iron infusion’ (Female, 55 years, Control—Placebo)
	Perceived effects of therapeutic treatment received	‘I have more energy, now whether that's to do with the attitude or from a large set of iron into my bloodstream—I don't know … I suppose that's the only thing—is my mind—or is it my mind playing tricks on me and after sort of six months am I going to start feeling tired again and will that because the iron's not in my bloodstream anymore, or will it be because my mind is playing tricks on me’ (Female, 53 years, Control—Placebo)
		‘It doesn't feel as much of a battle and as a struggle at all, which is amazing really, it's not—life doesn't seem as hard with the tiredness, it doesn't, it doesn't seem as difficult and I feel like I've, I don't know, got more in the tank, you know, whereas before things would probably rile me or I'd probably react in a certain way, whereas I feel like now I've just got a bit more perspective and just a bit more clarity about everything’ (Female, 38 years, Control—Placebo)
	Increased energy levels	‘The benefits are I feel better. I'm more well in myself. I feel much more energetic. I rest well so I'm more alert and my concentration is better … I've got—I don't know if clarity is the word—I just feel my concentration is better. Obviously when I feel better and I don't feel tired, yeah, I can concentrate better with work … Makes me feel good because at the end of that I still feel, I feel energetic, I feel like I'm—maybe psychological—I feel like I'm losing weight, I feel like, [laughs], you know, burning energy and losing weight’ (Female, 38 years, Intervention—Iron)
		‘I would say I was more alert than anything. I was up and ready to go, like, bustling about. … I was whizzing about, like, doing stuff, I was cleaning the house more, well I do clean the house as it is, but I don't let it get like dirty, I'm a clean freak, I have to—I've got a bit of an obsession, it's got to be clean’ (Female, 52 years, Control—Placebo)
Impact of trial participation on life participation	Beneficial effects of exercise programme	‘I found it loosened me up and I was able to move more freely after it. I definitely noticed a difference … perhaps I should exercise more because it did loosen me up and it did help—not with the pain—but it made the pain more bearable, it was less painful’ (Female, 55 years, Control—Placebo)
		‘For me personally the big stand out is the exercise programme, the resistance programme, which basically shows you where you are in terms of your—at the beginning, it shows you exactly where you are health wise, physical wise, strength wise—and as the programme went on, you could see the difference, the positives, which is, you know, compared to the beginning … the few weeks I was on the programme, I could definitely see, even the instructor could definitely see the change in my strength levels, my endurance levels going up, so yeah, that's the benefit that I had’ (Male, 62 years, Control—Placebo)
	Increasing understanding of own health, wellbeing, and lifestyle behaviours	‘Learnt more about my condition than I knew about it … More understanding of my muscle, how muscles work, and how it's all linked with everything else like the bones and muscles and things like that. That you need the muscles to work, you need the strength. If your muscles are weak you're not going to have the strength to do anything else and then you need your energy, you need the energy to build your muscles and be eating the right sort of food and things like that’ (Female, 57 years, Intervention—Iron)
		‘It taught me—in terms of—it just—to be honest it just made you aware of your condition you have and what you may need to do or what you should be doing … just the physical aspects of my life, it taught me to, just by doing a few hours of exercise a day, it can make you feel a lot more alert, a lot stronger and a lot mentally better … making me, made me feel about 40% better within myself’ (Male, 62 years, Control—Placebo)
	More positive outlook	‘I think I was on sort of a real downward spiral with my mental health … I just feel more up, you know what I mean, I don't feel like—I feel stronger in myself, in my body and in my mind, I feel like I've got more like clarity almost … I feel like now I've got more in me to do that, I feel like I've got my whole life to live’ (Female, 38 years, Control—Placebo)
		‘It made me feel like I wasn't a prisoner of kidney disease, I could actually take things into my own hands and make things better just by putting in a bit of effort really … I really kind of felt that I had really felt for a long time that I've got this kidney disease and I can't do anything about it. It is what it is. I can't cure it, I can't make it better. Whatever I do it's not going to change it. And actually it kind of proved something to myself that actually it doesn't have to define me … I kind of think it has made me more positive and given me some sort of motivation’ (Female, 59 years, Control—Placebo)
		‘This has been life changing already. And it really has been such a positive thing for me … I think for me it has made me realise that I am not as, I'm not as disabled as I thought I was by kidney disease … I was kind of putting up mental barriers thinking well you better not do this because you're an ill person … It basically made me realise that I don't have to define myself by being an ill person and I'm actually not that ill—whatever that means!’ (Female, 53 years, Control—Placebo)
	Increased functional performance	‘I can feel and see muscles building, like my leg muscles are much stronger, because when I manage to go, to fit my own regular gym sessions in, I do a lot better, yeah. I'm stronger. I know I'm stronger because I can feel it’ (Female, 38 years, Intervention—Iron)
		‘I can notice when I walk up the stairs. I still can't run upstairs four or five times, but I can zip up the stairs and down and do what I want … it used to be like one step at a time. Now I can take two steps at a time … I'm nowhere near as tired or exhausted as doing everyday things’ (Male, 63 years, Control—placebo)
		‘It's little things you're doing like whatever you're doing, say lifting or moving something or walking or just anything, it helps because it's made your body physically fitter, so you're not really getting out of breath and you're not feeling after you've done it, you're not feeling weary and tired. So in that way I think it's improved you physically that way and you just feel within yourself better that you can cope with a lot of things’ (Male, 62 years, Control—placebo)
	Changes in sleep quality and daytime sleepiness	‘I still nap in the afternoon at the moment, yeah, because—in the afternoon I do for about 10, 15 minutes. Before I used to nap quite a lot, I think, compared to now. And for less time as well now’ (Male, 51 years, Intervention—Iron)
		‘Sometimes I do have an afternoon nap … I think I choose to have a nap now, whereas before I would think oh god, I'’m so tired, I need to lie down and just collapse. Now I think well I haven't got anything to do, I've had a long walk, I think I'll just have forty winks before I carry on with my day’ (Female, 53 years, Control—Placebo)
		‘I tend to find myself going to sleep in the evenings … just drifting off to sleep for an hour/hour and a half … if I sit down in the early evening in an easy chair I will nod off. I never used to’ (Male, 65 years, Intervention—Iron)

GP, general practitioner.

#### Lack of awareness of iron deficiency prior to trial involvement

Most participants were unaware that they were iron deficient prior to being approached to take part in the trial. Some participants reported previously being prescribed iron tablets for a short period of time, in relation to another health event or state. There was a lack of understanding of iron deficiency and the potential causes of low iron, with participants reporting a scarcity of information available and limited discussions with their doctor. After learning that they had iron deficiency upon entry to the study, participants described what they perceived to be potential consequences of having low iron and the associated symptoms of living with CKD and iron deficiency.

#### Overwhelming feelings of tiredness

Overwhelming feelings of tiredness, including characteristics and feelings of physical and mental tiredness, were described prior to trial involvement. Participants tried to attribute reasons or causes for the tiredness they experienced; some perceived poor sleep and overexerting themselves as potential causes, whereas others felt there was no rationale. Poor sleep, including reduced sleep duration and disturbances due to symptoms (e.g. need for urination, muscle cramps, restless legs and pain), was considered to exacerbate feelings of tiredness, with many feeling that they were not well rested even after a good night's sleep. Participants believed that they were more in tune with their bodies and willing to respond accordingly rather than resist, with many employing energy management techniques (e.g. pacing of activities, having a more relaxed approach, or allowing for rest days) to help manage and minimize the tiredness experienced.

#### Feeling limited

Participants described feeling limited and self-conscious about their ability to perform tasks and activities. Increasing age, lack of physical strength and tiredness were perceived to be contributing factors to reduced functional ability. Some participants reported needing ‘rest and recharge’ days after engaging in social activities or physical hobbies, whilst others reported desisting from hobbies as they felt unable to continue them. Social activities were impacted, resulting in feelings of loneliness which were considered to be magnified by not having someone who understood and was able to relate to their experiences. Difficulties in staying alert, poor levels of concentration and memory were reported with participants finding it difficult to remember or recall information. A discordance between the participant's mind and body was frequently experienced, with beliefs around performing certain tasks but an inability to complete them as desired, impacting upon activities of daily living. Completing everyday tasks and activities was considered to require some level of motivation and determination.

#### Balancing emotions

Living with CKD was considered to be emotionally challenging, with participants frequently experiencing changes in mood and intense negative emotions. Participants described how there was discordance between how they felt and how they think they should feel; managing this conflict, and the desire to differentiate between tiredness and low mood, were challenges participants experienced daily. Overwhelming feelings of anger, frustration and concern were commonly reported. The emotional and psychological burden was challenging, with participants finding it difficult to cope and manage the consequences and impact, dispersing across all aspects of their lives.

#### Perceptions and experiences of therapeutic treatment received

There were mixed perceptions amongst participants about which therapeutic treatment (iron therapy or placebo) they had received, with no noticeable differences between groups. Several participants suspected that they received the treatment that they were not assigned to—some placebo group participants thought they received iron therapy, and some iron therapy group participants thought they received the placebo. Several participants changed their minds about what they thought they had received, believing initially to have received one treatment to then thinking they received the alternative treatment. Participants reported feeling and observing changes, both physically and mentally, which influenced their perceptions as to what treatment they received. For most participants, regardless of treatment received, symptoms were perceived to be alleviated following the infusion but were now returning. Changes in tiredness experienced were described, alongside increases in perceived sense of control over feelings of tiredness and its impact. Participants, including some who received the placebo, reported improvements in energy levels, ability to perform activities, concentration and sleep quality.

#### Impact of trial participation on life participation

Participants described the beneficial effects of participating in the trial, specifically the exercise training and improvements experienced as a result. The exercise training was reported to be initially challenging and exhausting; over time it was viewed more positively, especially when noticing changes. Physical improvements, including changes to body shape, strength and functional performance, were reported, along with improvements in mood and wellbeing. Study participation was perceived to increase participants’ understanding of their own health, wellbeing and lifestyle behaviours. Increased motivation and wellbeing were frequently discussed, with participants having a more positive outlook on life with CKD. Sleep quality was generally thought to have improved, and whilst some participants reported still napping during the day, daytime sleepiness was reduced.

## DISCUSSION

Our findings highlight a lack of awareness and understanding about iron deficiency and fatigue management among people living with CKD and iron deficiency. The effects and impact of tiredness experienced by the trial participants and their daily lives are profound, including reduced vitality and impaired ability to perform daily activities. Low mood and overwhelming feelings were frequently reported as consequences of emotional conflict. There were no clear differences reported between treatment groups, with mixed perceptions about the therapeutic treatment received. Study participation, specifically the exercise training, was perceived to be beneficial with improvements in life participation and psychological wellbeing experienced.

Participants were unaware of their iron deficiency and lacked understanding about the potential impact. Despite this, all participants reported experiencing overwhelming feelings of tiredness and substantial psychosocial and physical burden associated with living with CKD. Iron deficiency is often overlooked in people with long-term conditions, like CKD, as its most commonly related symptoms (e.g. fatigue, sleepiness) are not specific and often difficult to differentiate from symptoms of the primary disease [28]. Regardless of the underlying cause, symptoms experienced can negatively impact individuals’ lives and ability to engage in meaningful activities. Fatigue, or ‘feeling tired’, is a commonly reported debilitating symptom in those with CKD [29]; individuals with CKD are two to five times more likely to experience fatigue than those without CKD [30]. Muscle weakness or poor endurance, a sense of increased effort, and diminished cognitive endurance are also frequently experienced [31]. Fatigue is multifactorial and can manifest differently between individuals [32], consequently impairing their daily functioning, motivation and social engagement [33–38], and contributing to poor sleep quality and increased body pain [37–39]. Given the significance of fatigue and its consequences on patient outcomes and QoL, timely and effective management of fatigue represents a clinical priority [40]. Strategies to address and support the management of fatigue, including early assessment, have the potential to indirectly improve associated symptoms like depression [41]. Improved communication and support for symptom management, specifically fatigue, may be needed to improve symptoms for people living with CKD. In addition, educational programs, for both patients and healthcare professionals, providing information about the condition, its impact on health-related QoL and daily activities, and management strategies may be required to improve understanding and active management [21].

Impaired life participation and changes in social and leisure activities, including impaired ability to perform them, were considered by participants to lead to sudden changes in mood and overwhelming emotions. Interpreting fatigue as uncontrollable and lasting, alongside dysfunctional thinking styles in response to fatigue (e.g. catastrophizing, symptom focusing or perceiving fatigue as signs of bodily damage), can lead to increased levels of anxiety and low mood [42]. Given the multifactorial nature of fatigue, there are several potential avenues for treatment of fatigue in CKD. Exercise, both aerobic and resistance training, has been shown to improve fatigue and exercise capacity [43, 44], as well as depression and anxiety symptoms in people with CKD [45]. Despite clinical practice guidelines providing recommendations for physical activity and exercise [46], the prevalence of physical activity in people with CKD is low and worsens with disease progression [47]. Previous studies have highlighted the need for patient education and counselling about the importance of exercise for people with CKD and how to embed physical activity into their daily lives [48]. Novel ways to facilitate the implementation of physical activity guidelines have been gaining interest as a potential solution to promote and deliver physical activity and reduce high levels of physical inactivity; one such intervention is Kidney Beam, designed to digitally deliver physical activity and wellbeing support for people with CKD [49], with demonstrated improvements in mental health and health-related QoL [50].

All participants in this study reported beneficial effects from the exercise training. The exercise programme was considered initially to be challenging and tiring, but by the end of the trial participants felt more energetic and mentally alert, and physically stronger and fitter. Participating in the trial was perceived to have profound physical and psychological impacts. Increased positive outlook, energy levels and functional ability were perceived to be a result of the therapeutic intervention received, either the iron therapy, exercise training or a combination. It has been proposed that combining exercise training with therapeutic treatments, like iron therapy, could target disease-related derangements in the oxygen transport chain and result in enhanced physiological adaptations to exercise [51], potentially improving the QoL and vitality of people living with CKD and non-anaemic iron deficiency. Whilst no differences were observed between the two groups in subjective and objective outcomes of the main trial [25], longer follow-up periods may be required to observe the translation of the perceived effects into the outcomes measured. In addition, a large RCT may be warranted to determine whether intravenous iron is beneficial in people living with non-dialysis CKD who are iron-deficient.

Whilst participants in this study reported both psychological and physical improvements from participating in the trial, it was not possible to differentiate or attribute improvements to specific components. It is possible that improvements were a result of iron therapy, placebo effect, exercise training, sense of achievement from exercise training participation, attention and sense of purpose from clinical trial involvement (including frequent contact with the research team), and/or a combination of some or all components. Additionally, participants reported other benefits including increased understanding of their own health, wellbeing and lifestyle behaviours. Indirect benefits gained, including increased knowledge, confidence, sense of purpose, connection and feelings of belonging, have been reported as facilitators of participating in clinical trials [52]. Given the reciprocal relationship between fatigue and physiological and psychological factors [53], an assessment of factors and where one might intervene is the initial step to improving the management of people with fatigue [32]. Using pharmacological and non-pharmacological treatment methods to address factors associated with fatigue may improve QoL [41]. The provision of kidney-specific tailored psychosocial and physical rehabilitation interventions has been recommended to improve outcomes for people with CKD [54]. As life participation and fatigue are top priorities for people living with CKD [55], interventions should include strategies to support psychosocial wellbeing, alongside participation in meaningful activities, to improve QoL. A recent consensus document from the European Anaemia of CKD Alliance proposed a move towards a more holistic, personalized and long-term approach, with the focus of treatment on improving QoL without increasing the risk of adverse cardiovascular events and tailoring management strategies to the needs of the individual [21]. In addition, a recent cross-sectional study of a cohort of CKD patients identified several modifiable factors associated with health-related QoL for consideration when developing and testing interventions that aim to improve health-related QoL [16]. The growing evidence base demonstrates the value of the patient's voice in their treatment and care.

### Strengths and limitations

A strength of the study is the number of participants interviewed pre- and post-intervention. However, the number of participants interviewed who received intravenous iron was smaller than those who received the placebo. This is partly due to the cessation of recruitment to the interview sub-study on the announcement of the COVID-19 pandemic. Participants were recruited using convenience sampling as the eligibility criteria for the main trial were extensive and thus was difficult to purposively select participants. Post-intervention interviews were conducted only at the end of the trial. Ideally, participants also would have been interviewed 4 weeks post-intervention, following therapeutic intervention but before participating in exercise training; however, it was considered an additional interview alongside other trial outcome measures would over-burden participants. Given the complex nature of the trial intervention, it is not possible to determine the contribution of the specific components to the beneficial effects perceived and/or experienced by participants; however, the use of qualitative methodology provides a greater understanding of participants’ experiences.

## CONCLUSION

This qualitative study has highlighted that people with CKD and iron deficiency may experience substantial psychosocial and physical burdens. Most participants, including those who received the placebo, reported increased energy levels, ability to perform activities, concentration and sleep quality. Improved communication and support about the psychosocial impact and management of symptoms, particularly fatigue, for individuals with CKD may be required, alongside effective therapeutic interventions with exercise components to improve symptoms and QoL.

## Data Availability

The data that support the findings of this study are available from the corresponding author upon reasonable request.
